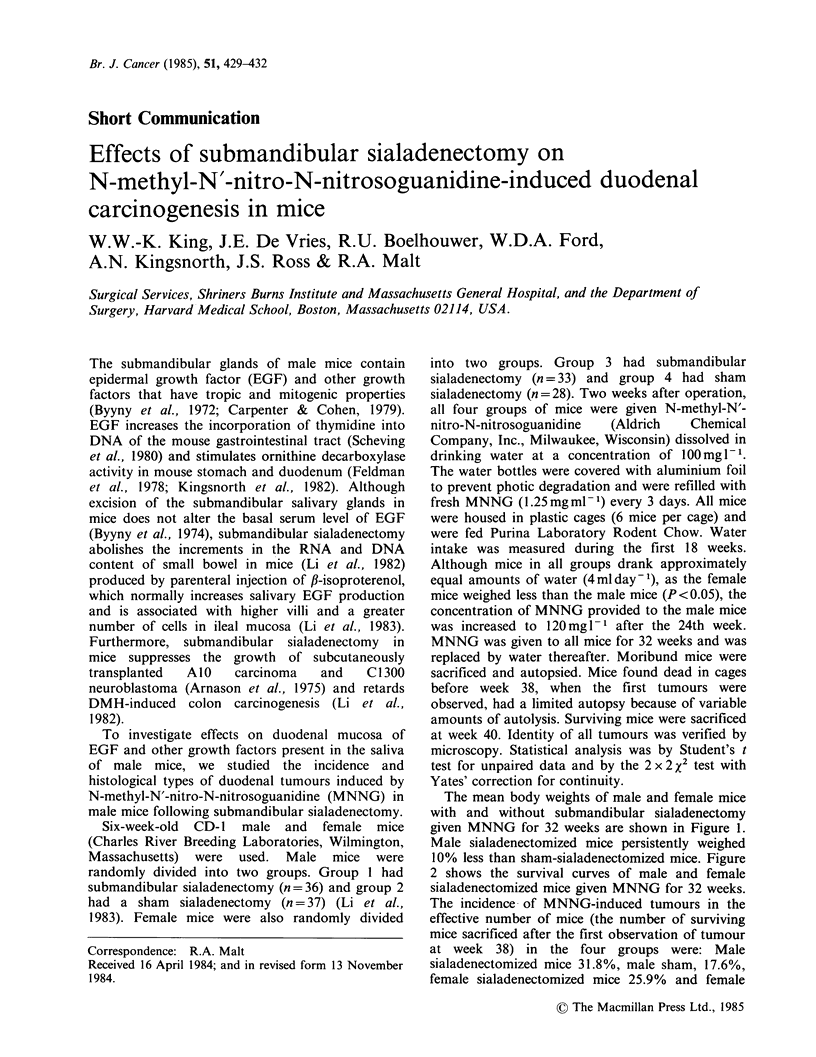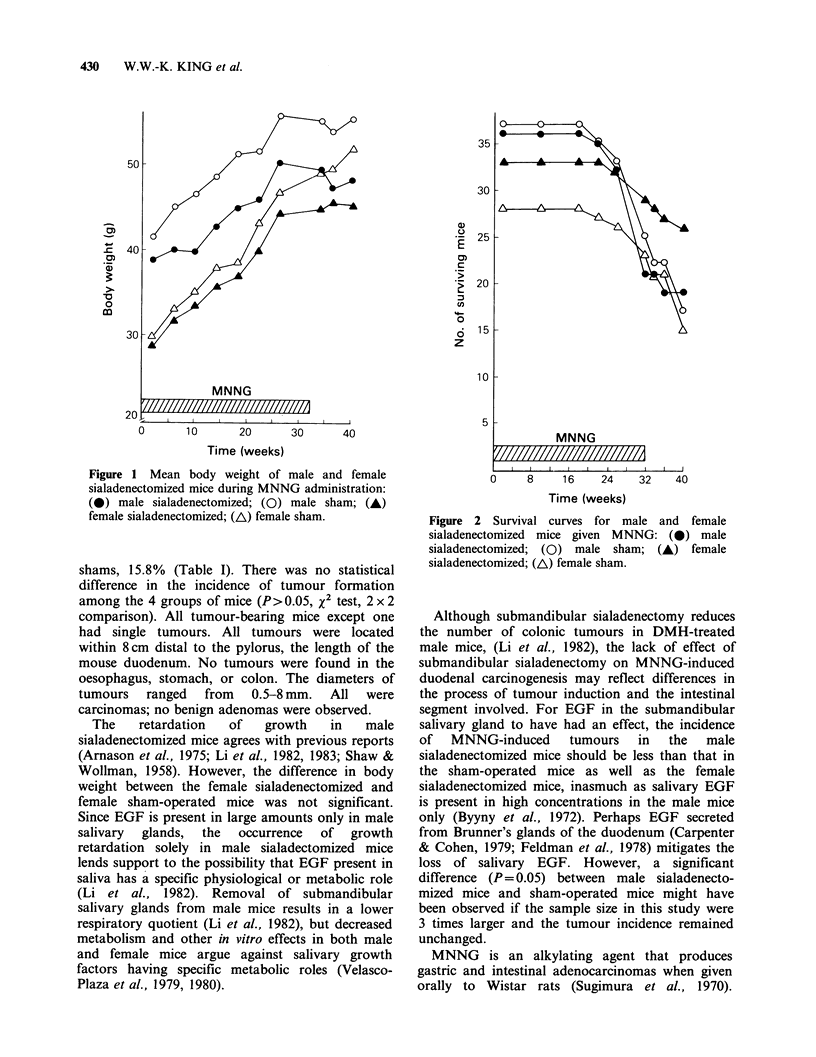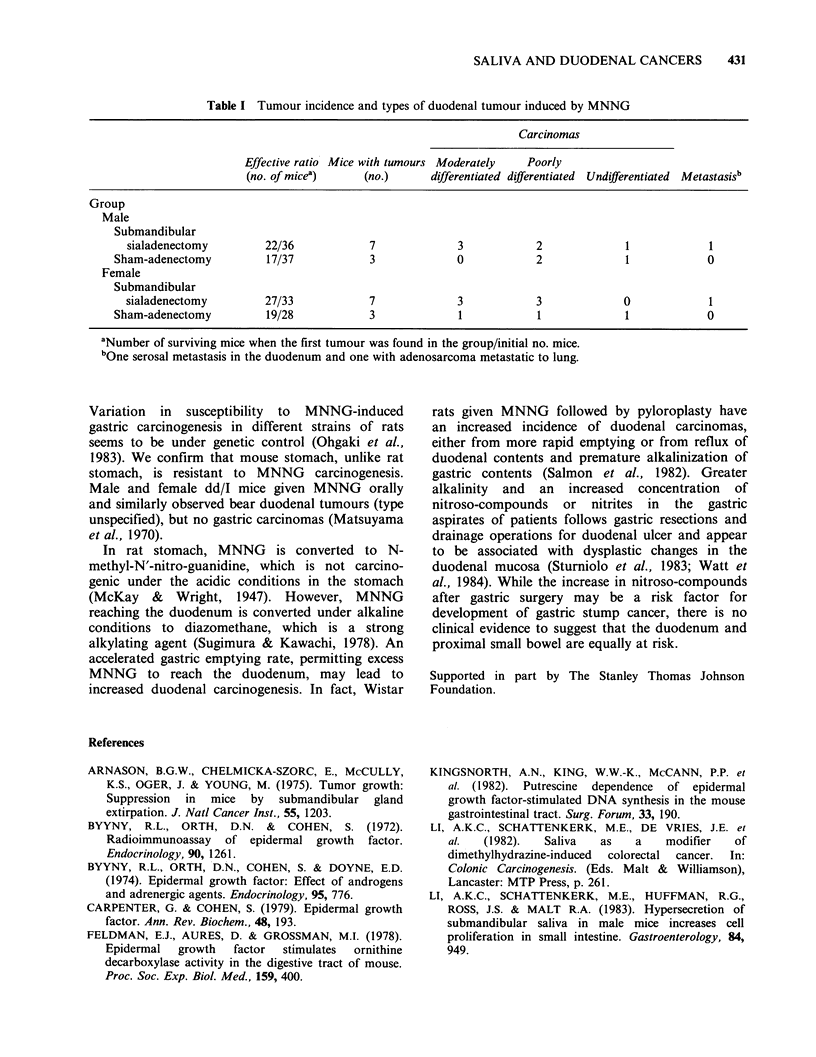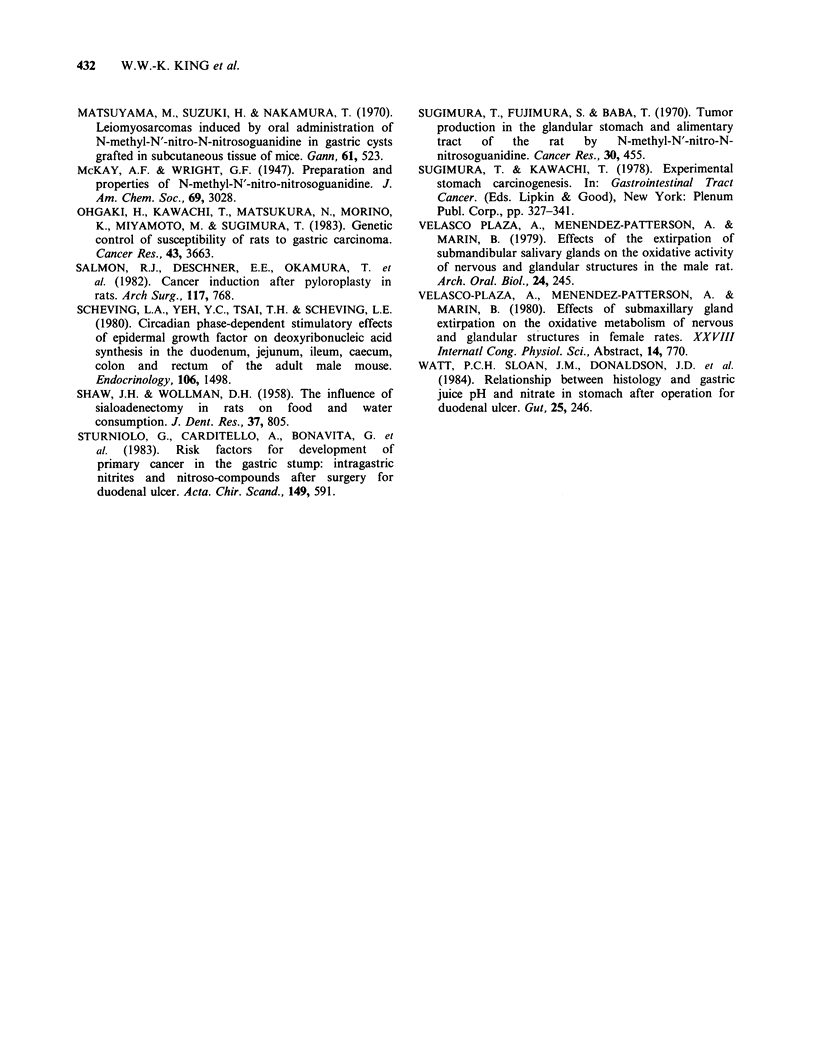# Effects of submandibular sialadenectomy on N-methyl-N'-nitro-N-nitrosoguanidine-induced duodenal carcinogenesis in mice.

**DOI:** 10.1038/bjc.1985.59

**Published:** 1985-03

**Authors:** W. W. King, J. E. De Vries, R. U. Boelhouwer, W. D. Ford, A. N. Kingsnorth, J. S. Ross, R. A. Malt


*Br. J. Cancer* (1985), **51**, 429–432

**Short Communication**

### Effects of submandibular sialadenectomy on N-methyl-N′-nitro-N-nitrosoguanidine-induced duodenal carcinogenesis in mice

W.W.-K. King, J.E. De Vries, R.U. Boelhouwer, W.D.A. Ford, A.N. Kingsnorth, J.S. Ross & R.A. Malt

*Surgical Services, Shriners Burns Institute and Massachusetts General Hospital, and the Department of Surgery, Harvard Medical School, Boston, Massachusetts 02114, USA.*

The submandibular glands of male mice contain epidermal growth factor (EGF) and other growth factors that have tropic and mitogenic properties (Byyny *et al.,* 1972; Carpenter & Cohen, 1979). EGF increases the incorporation of thymidine into DNA of the mouse gastrointestinal tract (Scheving *et al.,* 1980) and stimulates ornithine decarboxylase activity in mouse stomach and duodenum (Feldman *et al.,* 1978; Kingsnorth *et al.,* 1982). Although excision of the submandibular salivary glands in mice does not alter the basal serum level of EGF (Byyny *et al.,* 1974), submandibular sialadenectomy abolishes the increments in the RNA and DNA content of small bowel in mice (Li *et al.,* 1982) produced by parenteral injection of $\beta$-isoproterenol, which normally increases salivary EGF production and is associated with higher villi and a greater number of cells in ileal mucosa (Li *et al.,* 1983). Furthermore, submandibular sialadenectomy in mice suppresses the growth of subcutaneously transplanted A10 carcinoma and C1300 neuroblastoma (Arnason *et al.,* 1975) and retards DMH-induced colon carcinogenesis (Li *et al.,* 1982).

To investigate effects on duodenal mucosa of EGF and other growth factors present in the saliva of male mice, we studied the incidence and histological types of duodenal tumours induced by N-methyl-N′-nitro-N-nitrosoguanidine (MNNG) in male mice following submandibular sialadenectomy.

Six-week-old CD-1 male and female mice (Charles River Breeding Laboratories, Wilmington, Massachusetts) were used. Male mice were randomly divided into two groups. Group 1 had submandibular sialadenectomy ($n=36$) and group 2 had a sham sialadenectomy ($n=37$) (Li *et al.,* 1983). Female mice were also randomly divided into two groups. Group 3 had submandibular sialadenectomy ($n=33$) and group 4 had sham sialadenectomy ($n=28$). Two weeks after operation, all four groups of mice were given N-methyl-N′-nitro-N-nitrosoguanidine (Aldrich Chemical Company, Inc., Milwaukee, Wisconsin) dissolved in drinking water at a concentration of $100\,mg\,l^{-1}$. The water bottles were covered with aluminium foil to prevent photic degradation and were refilled with fresh MNNG ($1.25\,mg\,ml^{-1}$) every 3 days. All mice were housed in plastic cages (6 mice per cage) and were fed Purina Laboratory Rodent Chow. Water intake was measured during the first 18 weeks. Although mice in all groups drank approximately equal amounts of water ($4\,ml\,day^{-1}$), as the female mice weighed less than the male mice ($P<0.05$), the concentration of MNNG provided to the male mice was increased to $120\,mg\,l^{-1}$ after the 24th week. MNNG was given to all mice for 32 weeks and was replaced by water thereafter. Moribund mice were sacrificed and autopsied. Mice found dead in cages before week 38, when the first tumours were observed, had a limited autopsy because of variable amounts of autolysis. Surviving mice were sacrificed at week 40. Identity of all tumours was verified by microscopy. Statistical analysis was by Student's $t$ test for unpaired data and by the $2\times2\,\chi^2$ test with Yates' correction for continuity.

The mean body weights of male and female mice with and without submandibular sialadenectomy given MNNG for 32 weeks are shown in Figure 1. Male sialadenectomized mice persistently weighed 10% less than sham-sialadenectomized mice. Figure 2 shows the survival curves of male and female sialadenectomized mice given MNNG for 32 weeks. The incidence of MNNG-induced tumours in the effective number of mice (the number of surviving mice sacrificed after the first observation of tumour at week 38) in the four groups were: Male sialadenectomized mice 31.8%, male sham, 17.6%, female sialadenectomized mice 25.9% and female

Correspondence: R.A. Malt
Received 16 April 1984; and in revised form 13 November 1984.

© The Macmillan Press Ltd., 1985

**Figure 1** Mean body weight of male and female sialadenectomized mice during MNNG administration: (●) male sialadenectomized; (○) male sham; (▲) female sialadenectomized; (△) female sham.

**Figure 2** Survival curves for male and female sialadenectomized mice given MNNG: (●) male sialadenectomized; (○) male sham; (▲) female sialadenectomized; (△) female sham.

shams, 15.8% (Table I). There was no statistical difference in the incidence of tumour formation among the 4 groups of mice ($P>0.05$, $\chi^2$ test, $2\times2$ comparison). All tumour-bearing mice except one had single tumours. All tumours were located within 8 cm distal to the pylorus, the length of the mouse duodenum. No tumours were found in the oesophagus, stomach, or colon. The diameters of tumours ranged from 0.5–8 mm. All were carcinomas; no benign adenomas were observed.

The retardation of growth in male sialadenectomized mice agrees with previous reports (Arnason *et al.*, 1975; Li *et al.*, 1982, 1983; Shaw & Wollman, 1958). However, the difference in body weight between the female sialadenectomized and female sham-operated mice was not significant. Since EGF is present in large amounts only in male salivary glands, the occurrence of growth retardation solely in male sialadectomized mice lends support to the possibility that EGF present in saliva has a specific physiological or metabolic role (Li *et al.*, 1982). Removal of submandibular salivary glands from male mice results in a lower respiratory quotient (Li *et al.*, 1982), but decreased metabolism and other *in vitro* effects in both male and female mice argue against salivary growth factors having specific metabolic roles (Velasco-Plaza *et al.*, 1979, 1980).

Although submandibular sialadenectomy reduces the number of colonic tumours in DMH-treated male mice, (Li *et al.*, 1982), the lack of effect of submandibular sialadenectomy on MNNG-induced duodenal carcinogenesis may reflect differences in the process of tumour induction and the intestinal segment involved. For EGF in the submandibular salivary gland to have had an effect, the incidence of MNNG-induced tumours in the male sialadenectomized mice should be less than that in the sham-operated mice as well as the female sialadenectomized mice, inasmuch as salivary EGF is present in high concentrations in the male mice only (Byyny *et al.*, 1972). Perhaps EGF secreted from Brunner's glands of the duodenum (Carpenter & Cohen, 1979; Feldman *et al.*, 1978) mitigates the loss of salivary EGF. However, a significant difference ($P=0.05$) between male sialadenectomized mice and sham-operated mice might have been observed if the sample size in this study were 3 times larger and the tumour incidence remained unchanged.

MNNG is an alkylating agent that produces gastric and intestinal adenocarcinomas when given orally to Wistar rats (Sugimura *et al.*, 1970).

**Table I** Tumour incidence and types of duodenal tumour induced by MNNG

| | *Effective ratio (no. of mice[a])* | *Mice with tumours (no.)* | *Carcinomas* Moderately differentiated | *Carcinomas* Poorly differentiated | *Carcinomas* Undifferentiated | *Metastasis[b]* |
|---|---|---|---|---|---|---|
| Group | | | | | | |
| Male | | | | | | |
| Submandibular sialadenectomy | 22/36 | 7 | 3 | 2 | 1 | 1 |
| Sham-adenectomy | 17/37 | 3 | 0 | 2 | 1 | 0 |
| Female | | | | | | |
| Submandibular sialadenectomy | 27/33 | 7 | 3 | 3 | 0 | 1 |
| Sham-adenectomy | 19/28 | 3 | 1 | 1 | 1 | 0 |

[a]Number of surviving mice when the first tumour was found in the group/initial no. mice.
[b]One serosal metastasis in the duodenum and one with adenosarcoma metastatic to lung.

Variation in susceptibility to MNNG-induced gastric carcinogenesis in different strains of rats seems to be under genetic control (Ohgaki *et al.*, 1983). We confirm that mouse stomach, unlike rat stomach, is resistant to MNNG carcinogenesis. Male and female dd/I mice given MNNG orally and similarly observed bear duodenal tumours (type unspecified), but no gastric carcinomas (Matsuyama *et al.*, 1970).

In rat stomach, MNNG is converted to N-methyl-N′-nitro-guanidine, which is not carcinogenic under the acidic conditions in the stomach (McKay & Wright, 1947). However, MNNG reaching the duodenum is converted under alkaline conditions to diazomethane, which is a strong alkylating agent (Sugimura & Kawachi, 1978). An accelerated gastric emptying rate, permitting excess MNNG to reach the duodenum, may lead to increased duodenal carcinogenesis. In fact, Wistar rats given MNNG followed by pyloroplasty have an increased incidence of duodenal carcinomas, either from more rapid emptying or from reflux of duodenal contents and premature alkalinization of gastric contents (Salmon *et al.*, 1982). Greater alkalinity and an increased concentration of nitroso-compounds or nitrites in the gastric aspirates of patients follows gastric resections and drainage operations for duodenal ulcer and appear to be associated with dysplastic changes in the duodenal mucosa (Sturniolo *et al.*, 1983; Watt *et al.*, 1984). While the increase in nitroso-compounds after gastric surgery may be a risk factor for development of gastric stump cancer, there is no clinical evidence to suggest that the duodenum and proximal small bowel are equally at risk.

Supported in part by The Stanley Thomas Johnson Foundation.

### References

ARNASON, B.G.W., CHELMICKA-SZORC, E., McCULLY, K.S., OGER, J. & YOUNG, M. (1975). Tumor growth: Suppression in mice by submandibular gland extirpation. *J. Natl Cancer Inst.*, **55**, 1203.

BYYNY, R.L., ORTH, D.N. & COHEN, S. (1972). Radioimmunoassay of epidermal growth factor. *Endocrinology*, **90**, 1261.

BYYNY, R.L., ORTH, D.N., COHEN, S. & DOYNE, E.D. (1974). Epidermal growth factor: Effect of androgens and adrenergic agents. *Endocrinology*, **95**, 776.

CARPENTER, G. & COHEN, S. (1979). Epidermal growth factor. *Ann. Rev. Biochem.*, **48**, 193.

FELDMAN, E.J., AURES, D. & GROSSMAN, M.I. (1978). Epidermal growth factor stimulates ornithine decarboxylase activity in the digestive tract of mouse. *Proc. Soc. Exp. Biol. Med.*, **159**, 400.

KINGSNORTH, A.N., KING, W.W.-K., McCANN, P.P. *et al.* (1982). Putrescine dependence of epidermal growth factor-stimulated DNA synthesis in the mouse gastrointestinal tract. *Surg. Forum*, **33**, 190.

LI, A.K.C., SCHATTENKERK, M.E., DE VRIES, J.E. *et al.* (1982). Saliva as a modifier of dimethylhydrazine-induced colorectal cancer. In: *Colonic Carcinogenesis*. (Eds. Malt & Williamson), Lancaster: MTP Press, p. 261.

LI, A.K.C., SCHATTENKERK, M.E., HUFFMAN, R.G., ROSS, J.S. & MALT R.A. (1983). Hypersecretion of submandibular saliva in male mice increases cell proliferation in small intestine. *Gastroenterology*, **84**, 949.

MATSUYAMA, M., SUZUKI, H. & NAKAMURA, T. (1970). Leiomyosarcomas induced by oral administration of N-methyl-N′-nitro-N-nitrosoguanidine in gastric cysts grafted in subcutaneous tissue of mice. *Gann*, **61**, 523.

McKAY, A.F. & WRIGHT, G.F. (1947). Preparation and properties of N-methyl-N′-nitro-nitrosoguanidine. *J. Am. Chem. Soc.*, **69**, 3028.

OHGAKI, H., KAWACHI, T., MATSUKURA, N., MORINO, K., MIYAMOTO, M. & SUGIMURA, T. (1983). Genetic control of susceptibility of rats to gastric carcinoma. *Cancer Res.*, **43**, 3663.

SALMON, R.J., DESCHNER, E.E., OKAMURA, T. *et al.* (1982). Cancer induction after pyloroplasty in rats. *Arch Surg.*, **117**, 768.

SCHEVING, L.A., YEH, Y.C., TSAI, T.H. & SCHEVING, L.E. (1980). Circadian phase-dependent stimulatory effects of epidermal growth factor on deoxyribonucleic acid synthesis in the duodenum, jejunum, ileum, caecum, colon and rectum of the adult male mouse. *Endocrinology*, **106**, 1498.

SHAW, J.H. & WOLLMAN, D.H. (1958). The influence of sialoadenectomy in rats on food and water consumption. *J. Dent. Res.*, **37**, 805.

STURNIOLO, G., CARDITELLO, A., BONAVITA, G. *et al.* (1983). Risk factors for development of primary cancer in the gastric stump: intragastric nitrites and nitroso-compounds after surgery for duodenal ulcer. *Acta. Chir. Scand.*, **149**, 591.

SUGIMURA, T., FUJIMURA, S. & BABA, T. (1970). Tumor production in the glandular stomach and alimentary tract of the rat by N-methyl-N′-nitro-N-nitrosoguanidine. *Cancer Res.*, **30**, 455.

SUGIMURA, T. & KAWACHI, T. (1978). Experimental stomach carcinogenesis. In: *Gastrointestinal Tract Cancer*. (Eds. Lipkin & Good), New York: Plenum Publ. Corp., pp. 327–341.

VELASCO PLAZA, A., MENENDEZ-PATTERSON, A. & MARIN, B. (1979). Effects of the extirpation of submandibular salivary glands on the oxidative activity of nervous and glandular structures in the male rat. *Arch. Oral. Biol.*, **24**, 245.

VELASCO-PLAZA, A., MENENDEZ-PATTERSON, A. & MARIN, B. (1980). Effects of submaxillary gland extirpation on the oxidative metabolism of nervous and glandular structures in female rates. *XXVIII Internatl Cong. Physiol. Sci.*, Abstract, **14**, 770.

WATT, P.C.H. SLOAN, J.M., DONALDSON, J.D. *et al.* (1984). Relationship between histology and gastric juice pH and nitrate in stomach after operation for duodenal ulcer. *Gut*, **25**, 246.